# Health insurance in southeast Asia: is it enough for UHC?

**DOI:** 10.1016/j.lansea.2025.100653

**Published:** 2025-08-12

**Authors:** The Lancet Regional Health – Southeast Asia

One of the core strategies to achieve universal health coverage (UHC) is a health financing system that provides adequate financial protection to the population and is financed in an equitable and sustainable manner. Nations aim to provide universal access to quality health care while reducing financial hardship on their population. One important strategy that governments in several countries have adopted to achieve this goal is expanding publicly financed health insurance, which can reduce financial barriers to accessing health services, and protect against catastrophic health expenditures and impoverishment due to out-of-pocket expenditure.

Chancellor Bismarck is often credited with starting the first social health insurance system (‘sickness fund’) in the 19th century to provide workers with access to health care and social security. It gained momentum in 1930s and 40s in the USA and other countries, as employers promoted employer-based insurance as a way of attracting workers and managing health-care costs. Over the years, a plethora of health insurance schemes have emerged globally. They can present a diverse range of features depending on whether they are financed publicly (eg, through government taxes) or privately (eg, through individual premium contributions); whether contributions are mandatory (eg, payroll contributions for social insurance) or voluntary (eg, private insurance); whether they are managed by for-profit, not-for-profit, or government agencies; and whether services are provided by public or private providers. As an example, the Pradhan Mantri Jan Arogya Yojana (PM-JAY) insurance scheme in India is a tax-funded insurance scheme that provides free hospital care for a select set of services for people below the poverty line. Thailand’s Universal Coverage Scheme is also similarly tax-funded but covers inpatient and outpatient care for most of the population of Thailand. Smaller-scale health insurance schemes (ie, community-based insurance schemes) also exist in many countries, but they cover small geographies. Other schemes, such as microinsurance tailored for low-income populations, and workers’ compensation schemes to provide security for occupation-related injuries and illnesses, also fill a critical gap.

In countries of the WHO South-East Asia region (SEAR), health systems are at different stages of evolution and generally comprise public as well as private health-care providers. In countries such as India and Bangladesh, private providers account for 60–80% of outpatient visits and 40–60% of inpatient care. Out-of-pocket expenditure remains as high as 50% in some countries like India, Bangladesh, and Myanmar. While Indonesia and Bhutan provide broad coverage to their populations through social health insurance schemes, financial sustainability and inequities in access remain key challenges.

In this issue of *The Lancet Regional Health – Southeast Asia*, Singh and colleagues analyse data from Demographic and Health Surveys (2015–22) conducted in countries within SEAR. Their analysis finds stark disparities in coverage with regard to gender and socioeconomic and educational background; only one in five women in the region were covered by any form of health insurance, compared with one in four men; the highest coverage was observed in Indonesia (57%) and the lowest in Myanmar (1%). This study also highlighted that adults who had no formal education or lacked media exposure were less likely to be covered by any kind of insurance. People with higher educational attainment and within higher wealth quintiles were more likely to be covered by health insurance. The authors also found that, beyond individual-level factors, public insurance subscription was also influenced by contextual factors, such as government commitment, design and implementation of insurance schemes, and politico-economic conditions, as well as traditional beliefs and lack of trust in formal financial systems. These findings suggests that there is a need for more research into the macro-level factors rather than individual-based factors affecting enrolment and insurance uptake.

Publicly financed national health insurance programmes have the potential to impact equity and accessibility. However, it does not necessarily follow that the entire population will leverage these benefits. Many insurance schemes have restrictions, exclusions, or limited coverage for certain treatments, medications, or pre-existing conditions, and certain populations, which can leave large segments of the population, including many who are covered by these insurance programmes, vulnerable to high out-of-pocket costs. In particular, marginalised groups, people living with disabilities and rare conditions, informal sector workers, or those in rural areas may not fully benefit from these national publicly funded insurance programmes.

Azizatunnisa and colleagues analysed the Indonesia National Socioeconomic Survey and found that around 30% of people with disabilities were uninsured, and 35% were not enrolled, with coverage lower in the lowest socioeconomic groups, those living in rural areas, and self-employed people. However, people with disabilities utilised health-care services more frequently and incurred higher out-of-pocket and catastrophic health expenditures than those without disabilities. These findings highlight the fact that being enrolled or covered by an insurance programme does not necessarily provide financial protection. Further, policy makers should consider vulnerable populations while designing the policies. There should be more research into how the pooling mechanisms can account for fair and equitable resource allocation. There is a need for more research into barriers hindering vulnerable populations from insurance uptake. The field should invest in innovation through digital transformation, and should strive to enhance literacy among users and examine the patterns of implementation.

Achieving UHC is a fundamental aim of the UN’s Sustainable Development Goals. Although there are various other models of financing health care without putting citizens at risk of financial hardship, such as the National Health Service in the UK and public sector health services in Sri Lanka, many governments rely on health insurance as a potential strategy to meet the health-care and financial protection needs of their population. However, in many countries, these insurance programmes are still not adequately robust or effective. To realise the full potential of publicly financed health insurance programmes, governments need to strengthen regulation, embrace innovation, weed out inefficiencies and corruption, and engage with the implementation research community to understand the demand and supply side challenges that national insurance programmes experience.

